# Effect of Lockdowns on Hospital Staff in a COVID Center: A Retrospective Observational Study

**DOI:** 10.3390/vaccines10111847

**Published:** 2022-10-31

**Authors:** Giuseppe Vetrugno, Maurizio Sanguinetti, Rita Murri, Michela Sali, Simona Marchetti, Rosaria Santangelo, Massimo Fantoni, Antonella Cingolani, Giancarlo Scoppettuolo, Michele Di Donato, Vincenzo M. Grassi, Federica Foti, Luca Marchese, Fabio De-Giorgio, Antonio Oliva, Domenico Staiti, Francesco Maria De Simone, Domenico Pascucci, Fidelia Cascini, Roberta Pastorino, Denise Pires Marafon, Andrea Cambieri, Patrizia Laurenti, Stefania Boccia, Walter Ricciardi, Francesco Franceschi, on behalf of Gemelli-Against-COVID Group

**Affiliations:** 1 Fondazione Policlinico Universitario A Gemelli IRCCS, 00168 Rome, Italy; 2 Faculty of Medicine and Surgery, Università Cattolica del Sacro Cuore, 00168 Rome, Italy

**Keywords:** COVID-19, lockdown, swab, vaccination, healthcare workers

## Abstract

At the onset of the SARS-CoV-2 pandemic, individual and social measures were strengthened through restrictive non-pharmaceutical interventions, labelled with the term “lockdown”. In Italy, there were two lockdowns (9 March 2020–3 May 2020 and 3 November 2020–27 March 2021). As part of preventive measures, healthcare workers and the administrative staff population of Policlinico A. Gemelli underwent nasopharyngeal swab tests from 1 March 2020 to 9 February 2022, a long time interval that includes the two aforementioned lockdowns. The population included 8958 people from 1 March 2020 to 31 December 2020; 8981 people from 1 January 2021 to 31 December 2021; and 8981 people from 1 January 2022 to 9 February 2022. We then analysed pseudo-anonymized data, using a retrospective observational approach to evaluate the impact of the lockdown on the incidence of SARS-CoV-2 infections within the population. Given the 14 day contagious period, the swab positivity rate (SPR) among the staff decreased significantly at the end of the first lockdown, every day prior to 18 May 2020, by 0.093 (*p* < 0.0001, CI = (−0.138–−0.047)). After the fourteenth day post the end of the first lockdown (18 May 2020), the SPR increased daily at a rate of 0.024 (*p* < 0.0001, 95% CI = (0.013–0.034)). In addition, the SPR appeared to increase significantly every day prior to 17 November 2020 by 0.024 (*p* < 0.0001, CI = (0.013–0.034)). After the fourteenth day post the start of the second lockdown (17 November 2020), the SPR decreased daily at a rate of 0.039 (*p* < 0.0001, 95% CI = (−0.050–−0.027)). These data demonstrate that, in our Institution, the lockdowns helped to both protect healthcare workers and maintain adequate standards of care for COVID and non-COVID patients for the duration of the state of emergency in Italy.

## 1. Introduction

There is much evidence in the literature supporting the effectiveness of individual and social measures, such as wearing masks [1,2,3,4], hand washing [5], and physical/social distance [6], in reducing the spread of SARS-CoV-2 infections [7] and other respiratory viral infections [8]. After the introduction of vaccines, the Swiss cheese model has been used to show the need to employ all the available pandemic control measures to reduce the spread of the virus [9].

Other than this, there is little evidence supporting the validity of more restrictive non-pharmaceutical interventions (NPIs) that have been labeled with the term “lockdown”, such as the strict control of country borders and the closure of schools, workplaces, and other public institutions [10,11]. Some publications have even strongly questioned [12,13], if not actually denied, the efficacy of these solutions [14]. Alongside the purely health-related dimension of the lockdown measures, there has been a political dimension linked to the economic repercussions of NPIs; this has entailed the risk of debating about the efficacy or inefficacy of these solutions. From this perspective, the so-called Swedish model was initially thought to be indicative of the uselessness of NPIs [15], only to be proven less effective than originally thought [16].

In Italy, on 31 January 2020, the declaration of national emergency due to the COVID-19 pandemic became effective, and urgent measures to safeguard human health were implemented. On 23 February 2020, the so-called red zone (suspension of work and school activities and travel prohibition) was established in ten cities in Northern Italy. A lockdown was introduced for the first time on 9 March 2020, and subsequently extended until 3 May. Later, although in a less restrictive form, a second lockdown was declared on November 2020, which ended on 27 March 2021 [17].

The aim of this retrospective observational study is to assess the impact of the lockdown measures on the incidence of SARS-CoV-2 infections within a well-defined population, that is, the staff of a large hospital that was also a reference hub for the management of COVID-19 patients throughout the pandemic.

## 2. Materials and Methods

### 2.1. Setting

Policlinico A. Gemelli Hospital is both a welfare and educational institution. Eight clinical and research departments include 113 medical care units, including 86 complex operating units, 27 simple operating areas, and 1536 beds. In one year, 215 organ transplantations were performed, 94,509 patients were discharged, and 83,419 people accessed the emergency room

### 2.2. In-Hospital Protective Measures

On 25 February 2020, at Policlinico Gemelli Hospital, the Crisis Unit had its first meeting, determining all strategies for both health and administrative areas. On 28 February 2020, the red zone was extended to the Lombardy region. The following day, the first autochthonous case of COVID-19 in the Lazio region was diagnosed in our Institution. Within the hospital, several containment measures have been promptly introduced since March 2020, while our Institution was becoming a COVID center.

In order to manage both COVID and non-COVID hospitalized patients, the following two types of clinical pathways were created: the traditional one, reserved for negative patients, and one dedicated to confirmed cases of infection with SARS-CoV-2.

The staff population (see Supplementary Materials for stratification) has been protected by distribution of patients through these two different paths. The overlaps were limited by enhancing the use of remote working modes (smart working); modulating access to hospital dining areas; adopting personal protection measures (surgical masks initially, then FFP2 masks or higher in moderate risk contexts); implementing the policy on hand washing; improving compliance with behavioral safety standards (physical distancing, daily self-monitoring of temperature and concerning symptoms); and periodic monitoring and using a contact tracing system. These measures were enforced at the onset of the pandemic and strengthened over time [18].

**Figure 1:**
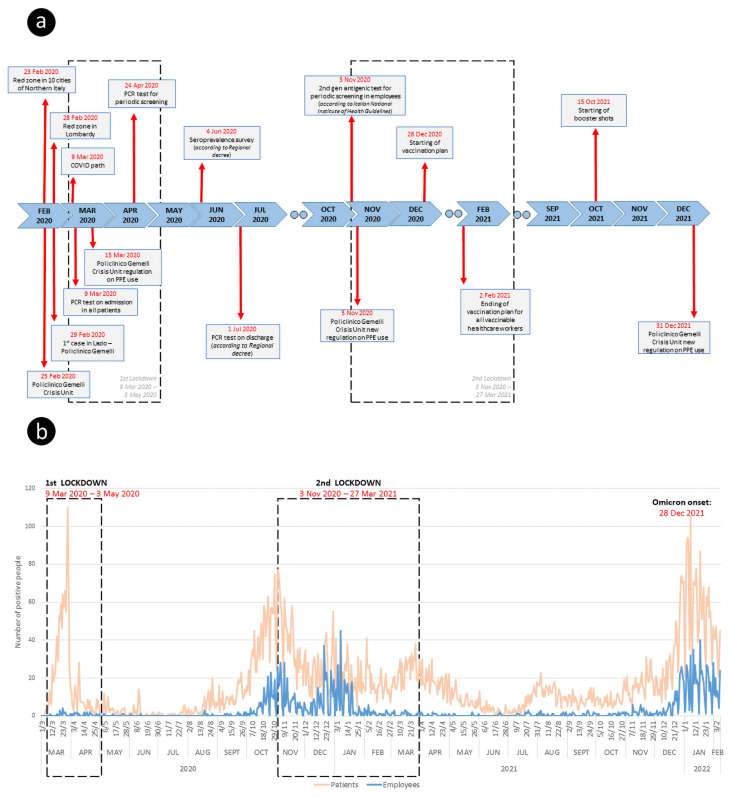
(**a**) Timeline representing both interventions based on National/Local Health Authorities and measures adopted in our Institution; (**b**) The trend of infections and positivity in the staff group and the patient group admitted to our Institution.

The above-described protection model was further strengthened by the vaccination plan [19] using the BNT162b21 vaccine, launched on 28 December 2020 and concluded in our Policlinic on 2 February 2021 for most of vaccinable healthcare workers, before the introduction of mandatory vaccination by the National Health Authority on 1 April 2021. At the end of the plan, the vaccination rate was as follows: medical staff, 90.7%, residents, 81%, technical staff, 90%, nurses and obstetricians, 85.2%, auxiliary staff, 87.1%. The timeline of these events is outlined in Figure 1a.

### 2.3. Population

This study is based on a staff population of healthcare workers (medical staff, nurses, and allied health professions), administrative and support staff, and a heterogeneous group including maintenance workers, food service workers, cleaners, etc. (see Supplementary Materials for details).

The staff population underwent nasopharyngeal swab tests from 1 March 2020 to 9 February 2022, alongside the patients, as mentioned in Table 1. All results were automatically sent to staff on their respective company e-mails, protected by a password separately received by SMS, and were collected and processed by the Hospital Occupational Health Service, with the support of software made available by the hospital’s data warehouse.

The ospital Occupational Health Service assesses the health status of employees in relation to the risks to which they are exposed. With respect to exposure to COVID risk, in accordance with a specific regional arrangement, the activity of Hospital Occupational Health Service has been enhanced with the support of the Risk Management Unit, which collects data on patients testing positive for COVID-19 through an automatic sending system updated every 12 h.

### 2.4. Patient and Public Involvement

It was not possible to involve the participants because the study was conducted retrospectively.

### 2.5. Statistical Analysis

All performed swabs were collected in the four weeks immediately before and after the start of the lockdown and were compared with those from the four weeks following the end of that period.

The number of positive people initially found and the positivity rate in the total number of swabs performed, distinguished by type of swab and cumulative number, were extracted from the database of our Institution for each population.

The positivity rate was also calculated as the average number of positive people recorded initially per week within the two populations. To limit possible confounding factors when calculating the positivity rate in the staff population, the days on which less than 10 swabs were carried out were removed from the calculation. The rationale for this cut-off relies on eliminating the following two confounding variables: a very small number of swabs were performed on healthcare workers during the initial days of the pandemic, due to equipment shortage; and the number of swabs was persistently ≤9 on holidays (Easter, Christmas, etc.) in the staff population during the following months. Data relating to admissions for infection with SARS-CoV-2 registered in the staff population in the period 1 March 2020–9 February 2022 and their outcome (discharge at home, transfer to other facilities, death) were also extracted.

Given the contagious period was calculated as 14 days in March 2020, the daily swab positivity ratio (SPR) was calculated and used as data points in the time series analysis using 23 March 2020 (start of first lockdown + 14 days), 18 May 2020 (end of first lockdown + 14 days), 17 November 2020 (start of second lockdown + 14 days) and 11 April 2021 (end of second lockdown + 14 days) as the intervention points.

Data were modelled as a single group interrupted time series analysis without a comparator for both scenarios using the *itsa* syntax in Stata^®^. The level and trajectory of change following the intervention, obtained via ordinary least-squares regression estimates, were evaluated yielding β-coefficients and Newey–West standard errors.

Statistical analysis was performed using Stata software (StataCorp. 2017. Stata Statistical Software: Release 15.1. StataCorp LP, College Station, TX, USA).

## 3. Results

From 1 March 2020 to 31 December 2020, the staff population consisted of 8958 people and a total of 36,437 swabs were performed, 22,148 molecular (M) and 14,289 antigenic (A). From 1 January 2021 to 31 December 2021, the staff population consisted of 8981 people, and a total of 105,783 swabs were performed (15,971 M and 89,812 A). Finally, from 1 January 2022 to 9 February 2022, the staff population consisted of 8981 people and 25,937 swabs were performed (4817 M and 21,120 A).

In the three above reported periods, the number of patients accepted were 113,842, 117,834, and 12,684, respectively. They underwent a total of 111,486 swabs (108,112 M and 3374 A) in the first period; 200,073 swabs (185,153 M and 14,920 A) in 2021; and 29,895 swabs (27,640 M and 2255 A) until 9 February 2022.

In the staff group, there were 40 reinfections between March 2020 and February 2022, with an average interval of 11 months between the first and second episode. The minimum interval between two newly acquired cases was 45 days and was observed from the beginning of January 2022; these re-infections were caused by the Omicron variant in previously Delta-infected subjects during November 2021. In the same group, a single fatality was recorded in a radiology technician in his fifties, who died in October 2020.

The trend of infections and positivity in the staff group and the patient group admitted to our Institution in the period 1 March 2020–9 February 2022 is shown in Figure 1b.

Table 2 summarizes the positivity rate in the staff and patient groups, segmented from the beginning of the first lockdown (9 March 2020) until the 14 day period following the end of the lockdown (17 May 2020), and from the 14 days period prior to the beginning of the second lockdown (21 October 2020) to the end of the first week following the conclusion of the vaccination campaign (administration of the second dose for most healthcare workers) on 9 February 2021. The time period from 23 September 2021 to 9 February 2022 was also used to compare the lockdown period with an interval without lockdown, but under the influence of the vaccination campaign which reached its maximum expansion through the use of the booster shot.

**Figure 2:**
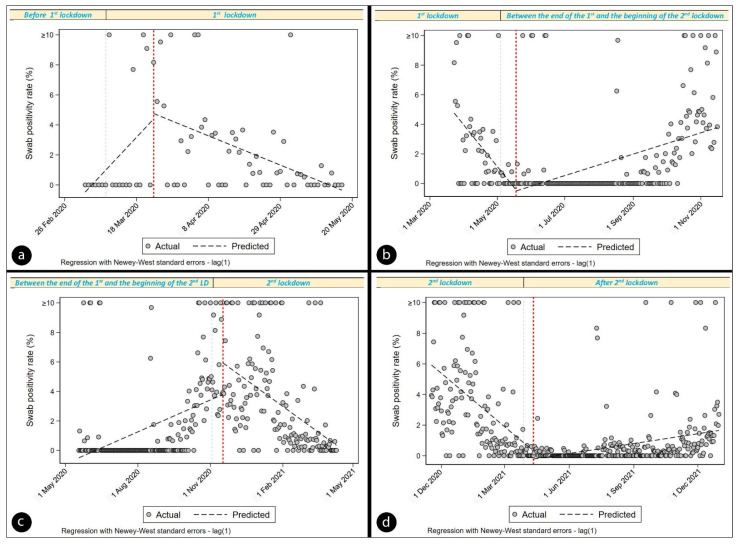
Regression with Newey–West standard errors. The red dotted line represents the intervention point. (**a**) shows postlinear trend regression output from 23 March 2020 (start of 1st lockdown + 14 days) intervention point. (**b**) represents the increase of swab positivity rate after the 14th day after the end of 1st lockdown (18 May 2020); (**c**) displays the decrease of swab positivity rate after the 14th day after start of 2nd lockdown (17 November 2020); (**d**) illustrates the increase of swab positivity after the 14th day after the end of 2nd lockdown (11 April 2021) at a rate of 0.007.

**Figure 3:**
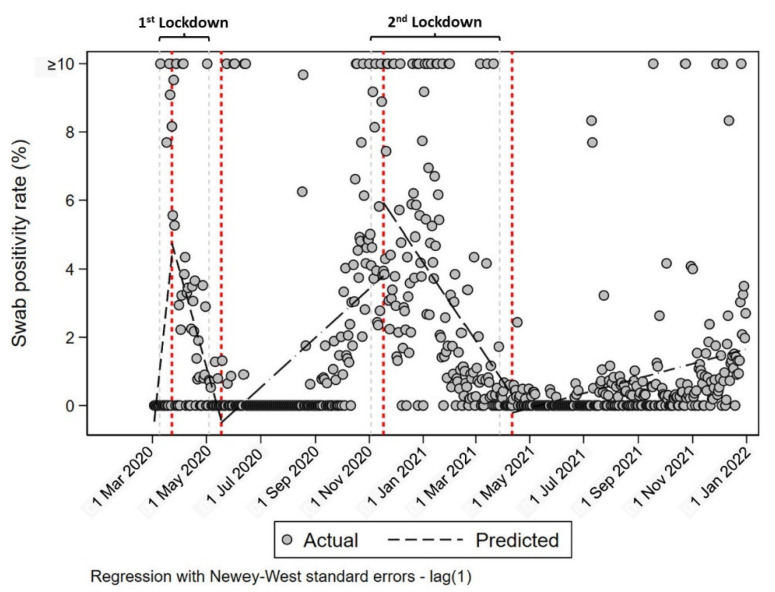
Daily swab positivity rate reported over time showing the intervention points. Red dotted lines represent the start and the end of each lockdown + 14 day.

Table 3, Table 4, Table 5 and Table 6 show the daily SPR, which was calculated using 23 Mar 2020 (start of first lockdown + 14 days); 18 May 2020 (end of first lockdown + 14 days); 17 November 2020 (start of second lockdown + 14 days); and 11 April 2021 (end of second lockdown + 14 days) as the intervention points (Figure 2 and Figure 3).

Table 3 shows interrupted time series ordinary least-squares regression output on the 23 March 2020 intervention point (period: 3 March 2020–17 May 2020) and post linear trend regression output from the 23 March 2020 intervention point.

As shown in the regression Table 4, SPR appeared to decrease significantly every day prior to 18 May 2020 by 0.093 (*p* < 0.0001, CI = (−0.138–−0.047)). In the first days of the intervention, there is no decrease, followed by a significant increase in the daily trend of SPR (relative to the preintervention trend) of 0.116 per day (*p* < 0.0001, CI = (0.070–0.163)). We also see, from the *lincom* estimate produced by specifying *posttrend*, that after the fourteenth day post the end of first lockdown (18 May 2020), the SPR increased daily at a rate of 0.024 (*p* < 0.0001, 95% CI = (0.013–0.034)).

As shown in the regression Table 5, SPR appeared to increase significantly every day prior to 17 November 2020 by 0.024 (*p* < 0.0001, CI = (0.013–0.034)). In the first day of the intervention, there appeared to be a significant increase of 2.130 (*p* = 0.008, CI = (0.550–3.711)) in SPR per day, followed by a significant decrease in the daily trend of SPR (relative to the preintervention trend) of 0.062 (*p* < 0.0001, CI = (−0.078–−0.046)). We also see, from the *lincom* estimate produced by specifying *posttrend*, that after the fourteenth day post the start of second lockdown (17 November 2020), the SPR decreased daily at a rate of 0.039 (*p* < 0.0001, 95% CI = (−0.050–−0.027)).

As shown in the regression Table 6, SPR appeared to decrease significantly every day prior to 11 April 2021 by 0.039 (*p* < 0.0001, CI = (−0.050–−0.027)). In the initial days of the intervention, there was no decrease, followed by a significant increase in the daily trend of SPR (relative to the preintervention trend) of 0.046 (*p* < 0.0001, CI = (0.034–0.058)). We also see, from the *lincom* estimate produced by specifying *posttrend*, that after the fourteenth day post the end of second lockdown (11 April 2021), the SPR increased daily at a rate of 0.007 (*p* < 0.0001, 95% CI = (0.004–0.010)).

## 4. Discussion

Most of the literature describing the effectiveness of restrictive measures during the pandemic emergency reflects the experience of countries that have been able to strengthen the strategic importance of their geographical position [10], or who had already developed capillary contact tracing systems and models of quarantine and isolation because of past pandemics [20].

Previous studies are based on estimation of the effectiveness of NPIs by the use of a statistical model to estimate the impact of each intervention on the incidence of COVID-19 [6]; other contributions were focused on mortality, data extracted from National Public Health Institutes [21], and characterized by large variability [22,23].

Moreover, several studies have shown that restrictive measures can lose their efficacy because of many factors in the medium and long term [24,25]. Nevertheless, this contribution, although based on a small sample, suggests that the adopted solutions have been able to limit the spread of the virus, even though the successful choice has been difficult. The Swedish approach has been highlighted several times, but has recently been severely criticized [16].

### 4.1. The Italian Context

During the first lockdown, the Italian government tried to limit the spread of the virus to Northern Italy and adopted a 24/7 curfew.

For the duration of the second lockdown, the curfew, ranging from 10 PM to 5 AM, was complementary to measures such as distance learning for high schools, closure of shopping malls on weekends, a reduction of 50% in the capacity of public transport, and the semi-closing of bars and restaurants from 6PM. The measures were then tightened during the Christmas holidays (from 21 December to 6 January).

While the first lockdown had an impact on a population of patients and healthcare workers who were less exposed to the virus because it was concentrated in Northern Italy, the second lockdown involved the same staff population and a different set of patients, at a time when the virus was widespread throughout the country.

### 4.2. Effect of Lockdowns on Hospital Staff

Given that the contagious period calculated as 14 days in March 2020 [26,27], it was expected that the influence on the positivity rates recorded should be substantial from the fifteenth day after the start of the lockdown.

We accordingly segmented the period of observation into segments of 28 days, starting from the first day of the first lockdown, to compare the rates of positivity recorded in the population that did not change during the period (the staff). We wanted to verify whether the lockdown measures have had a positive effect, in addition to the other protective measures adopted during the period and described in the Materials and Methods.

This verification has been provided during the following four periods: (1) the first lockdown (9 March–3 May 2020) and the 14 day period immediately following it, which represents the interval of the lockdown’s assumed effectiveness; (2) the period between the first and the second lockdown; (3) the second lockdown (2 November 2020–27 March 2021) which was different from the first, and influenced by the vaccination campaign; (4) the period without lockdown (14 April 2021–9 February 2022), when the proportion of the population who were vaccinated increased noticeably.

Regarding vaccinated healthcare workers, the protection period has been considered from 9 February 2021, as the majority of second doses were administered from 21 January 2021 to 2 February 2021, and the protective effect is expressed best after at least seven days from the date of administration of the second dose [28].

This effect can be observed in Figure 1b, which shows the positivity rates related to the first and the second lockdown, where the reduction 14 days after the start of the lockdown is visually represented. This effect is much more noticeable in the population of health workers than in the general population. Our data and Figure 1b also suggest that community-acquired infections considerably outweighed hospital-acquired infections.

In the patient group, the number of positive cases recorded on average fluctuated from 30.71 (in the two weeks from 9 March to 22 March 2020) to 24.61 (in the following four weeks from 23 March to 19 April 2020). This decreased even more significantly to the value of 3.68 in the next four weeks (from 20 April to 17 May 2020).

In the staff group, after a paradoxical increase recorded in the weeks from 23 March to 19 April 2020 (0.79 vs. 0.43 recorded in the previous weeks 9 March–22 March), we observed a decrease to 0.29 in the weeks from 20 April to 17 May 2020.

The trend is confirmed by data pertaining to the second lockdown, which is weaker, but in a population more exposed to the virus. Among patients, it went from a value of 50.93 in the period from 21 October to 17 November 2020, to the value of 25.36 in the period from 18 November to 15 December 2020. Among the staff population, it is interesting to compare the value of 11.32 in the period 21 October to 17 November 2020 to the value of 4.86 in the following period (18 November to 15 December 2020). Moreover, in January 2021 we observed that the two populations showed different patterns (Figure 1b), where the positivity rate of patients decreased slowly.

The effect seemed to persist over time, not only in the periods from 16 December 2020 to 12 January 2021, and from 13 January 2021 to 9 February 2021 (the conclusion of the vaccination campaign through the administration of the second dose for most of healthcare workers), but even in the same period of the following year (16 December 2021 to 9 February 2022).

In particular, considering that in our Institution we completed the administration of the second dose to most healthcare workers between 21 January and 2 February 2021 (and assuming the beginning of vaccine’s efficacy in the following seven days, starting from 9 February), there was a marked reduction of the positivity rate due to a synergistic effect of both the second lockdown and vaccination, together with a lower prevalence in the following months. We then registered a new increase in positivity rate, as a consequence of the following three factors: (1) a progressive decrease of the protective effect of the second dose, as has already been reported in literature [29,30], (2) the increase of the Delta variant in December 2021, and (3) the emergence of the Omicron variant between December 2021 and January 2022, in the absence of a new lockdown.

Moreover, in the staff population there was an average positivity rate of 11.89 during the lockdown period (16 December 2020–12 January 2021); this reflects a possible influence of both Christmas holidays and the spread of the Alpha variant. A value of 13.32 was recorded in the same period of the following year (16 December 2021–12 January 2022) in the absence of a lockdown. This trend is reinforced by the comparison between the period 13 January 2021–9 February 2021 (3.96) and the period 13 January 2022–9 February 2022 (14.79) in almost the same population.

The comparison between the data recorded during the first and second lockdown versus the remaining periods demonstrates that in our Institution these restrictive measures, together with the other classical preventive actions and, subsequently, with the synergistic effect of vaccination, helped not only to protect healthcare workers, but also to maintain adequate standards of care for COVID and non-COVID patients during the state of emergency in Italy.

Furthermore, our results could help to answer the question of what would have happened in Italy in the first period, if the lockdown had not been adopted, considering the limited percentage of vaccinated people.

## 5. Study Limitations

The limitation of this study lies in the long time interval covered, affected by the spread of the SARS-CoV-2 variants (Delta in addition to Alpha since March–April 2021, and Omicron in January 2022). Regarding the vaccination plan, while all vaccinable healthcare workers received their second dose by 2 February 2021, the vaccination for administrative staff began on 5 February 2021.

Another limitation is related to the accuracy of laboratory tests: since the beginning of the pandemic, the phenomenon of false negative responses even for molecular tests has been highlighted, especially in the period prior to the onset of symptoms [31]. This phenomenon seemed to be much more evident when using antigenic tests [32], particularly rapid lateral flow antigen detection tests [33]. In trying to overcome this inconvenience, the strategy implemented in our Institution was to periodically perform tests, according to the scheme reproduced in Table 1. The sensitivity and specificity of the tests used also contributed to limiting this phenomenon: the PCR test (Allplex TM SARS-CoV-2 Assay Seegene) has a sensitivity of 50 copies per reaction and specificity of 100%, while the antigenic test (Lumipulse G SARS-CoV-2 Antigen Fujirebio) has sensitivity of 92.5% and specificity of 100%. In any case, the substantial homogeneity of the staff population and the use of same type of test should make the impact of this possible confounding factor negligible.

Moreover, during the early part of the pandemic, most healthcare workers were less exposed to the virus, due to its greater concentration in Northern Italy [34] and adoption of the lockdown. Meanwhile, in the first weeks of lockdown, personal protective equipment (PPE) was not available for each healthcare worker. Due to increased consumption and the scarcity of PPE, the priority was to equip those workers who were primarily exposed.

Finally, the approach to pandemics has evolved from the first phase, characterized by the availability of molecular and serological swabs, although the latter proved to be poorly sensitive [35], to the introduction of antigenic testing with increasing sensitivity up to the fourth generation.

## Figures and Tables

**Table 1:** The staff population underwent nasopharyngeal swab tests from 1 March 2020 to 9 February 2022, alongside the patients.

STAFF						
1 March 2020–2 November 2020 PCR Test Only				3 November 2020–22 December 2021 Antigenic Test		23 December 2021–9 February 2022
1 March 2020–23 April 2020		24 April 2020–2 November 2020		3 November 2020–15 November 2020 2nd gen antigenic test *If >10 and <5000 pg/mL: confirmatory PCR*	16 November 2020–22 December 2021 3rd gen antigenic test *Positive if >10 pg/mL*	4th gen antigenic test *Positive if >10 pg/mL*
Not available		Periodic screening: Every 14 days, Emergency Department (ED) + COVID depts Every 30 days, ordinary depts Every 45 days, low risk depts and administrative staff Seroprevalence 4 June 2020–3 July 2020 (point-of-care testing—POCT + venous sample)		Periodic screening: Every 10 days, ED Every 14 days, COVID depts and high risk ordinary depts Every 21 days, moderate risk ordinary depts Every 35 days, low risk ordinary depts and administrative staff	Periodic screening: Every 10 days ED Every 14 days COVID depts Every 21 days ordinary depts Every 35 days others	Periodic screening: Every 10 days *
Contact tracing: Every 48 h from day 0 to 10 On day 14		Contact tracing: Every 48 h from day 0 to 14		Contact tracing: Every 48 h from day 0 to 10 On day 14	Contact tracing: Every 48 h from day 0 to 10	Contact tracing: On day 2 and 5
**PATIENTS**						
**1 March 2020–2 November 2020** PCR test				**3 November 2020–22 December 2021** Preliminary 2nd gen antigenic + PCR		**23 December 2021–9 February 2022**
1 March 2020–8 March 2020 PCR only	9 March 2020–3 May 2020 PCR only	4 May 2020–31 July 2020 Preliminary serologic POCT + PCR	1 August 2020–2 November 2020 Preliminary serologic POCT + PCR	3 November 2020–6 July 2021	7 July 2021–22 December 2021	Preliminary 3rd gen antigenic + PCR
Screening: On admission, only symptomatic with epidemiological link	Screening: On admission, all patients during hospitalization, symptomatic and asymptomatic with epidemiological link	Screening: Admission Transfer to health facilities	Screening: Admission Discharge	Screening: Admission Discharge	Screening: Admission Every 5 days Discharge	Screening: Admission On day 3 (only if admission from ED or other hospital) Every 5 days Discharge
Contact tracing: Every 48 h from day 0 to 14	Contact tracing: Every 48 h from day 0 to 14	Contact tracing: Every 48 h from day 0 to 14	Contact tracing: Every 48 h from day 0 to 10	Contact tracing: Every 48 h from day 0 to 10	Contact tracing: Every 48 h from day 0 to 10	Contact tracing: Every 48 h from day 0 to 10

* in accordance with a specific regional arrangement. Contact tracing: as a result of episodic positivity found in patients and/or employees.

**Table 2:** The positivity rate in the staff and patient population (the rows referring to the periods immediately before and after the lockdowns, considering the beginning of each lockdown + 14 days, are highlighted in bold).

Period			First Positivities/Day		Positivity Rates		
From	To	Days	Staff	Patients	Staff > 10 *	Staff Total	Patients
**9 March 2020**	**22 March 2020**	**14**	**0.43**	**30.71**	**9.23%**	**6.19%**	**18.46%**
**23 March 2020**	**19 April 2020**	**28**	**0.79**	**24.61**	**1.79%**	**1.77%**	**8.80%**
20 April 2020	17 May 2020	28	0.29	3.68	0.30%	0.30%	1.44%
**21 October 2020**	**17 November 2020**	**28**	**11.32**	**50.93**	**3.54%**	**3.53%**	**9.15%**
**18 November 2020**	**15 December 2020**	**28**	**4.86**	**25.36**	**1.73%**	**1.73%**	**4.95%**
16 December 2020	12 January 2021	28	11.89	24.36	3.41%	3.40%	5.25%
13 January 2021	9 February 2021	28	3.96	19.75	1.23%	1.22%	3.84%
23 September 2021	20 October 2021	28	0.43	8,18	0.16%	0.16%	1.51%
21 October 2021	17 November 2021	28	1.39	12.86	0.43%	0.43%	2.25%
18 November 2021	15 December 2021	28	1.46	17.96	0.41%	0.41%	3.00%
16 December 2021	12 January 2022	28	13.32	52.43	1.91%	1.91%	7.95%
13 January 2022	9 February 2022	28	14.79	46.43	2.61%	2.61%	7.34%

* The days on which less than 10 swabs were carried out on healthcare workers were removed from the calculation to eliminate possible confounding variables. The rows referring to the periods immediately before and after the lockdowns, considering the beginning of each lockdown + 14 days, are highlighted in bold (see table caption).

**Table 3:** Interrupted time series ordinary least-squares regression output at 23 March 2020 intervention point (period: 3 March 2020–17 May 2020). *N* = 76 (20 + 56).

	β-Coefficient	Std Error	*p* Value	95% CI
Preintervention ^1^	0.243	0.127	0.06	−0.010–0.497
Immediately postintervention ^2^	0.362	2.139	0.866	−3.902–4.626
Postintervention ^3^	−0.336	0.128	0.011	−0.591–−0.081
_cons ^4^	−0.474	0.979	0.63	−2.425–1.478
Postlinear trend regression output from 23 March 2020 intervention point.				
	β-Coefficient	Std error	*p* value	95% CI
Postintervention linear trend	−0.093	0.024	0.0002	−0.140–−0.046

^1^ Slope prior to intervention. ^2^ Change in level in the period immediately following intervention initiation (compared with counterfactual). ^3^ Difference between preintervention and postintervention slopes. ^4^ Intercept.

**Table 4:** Interrupted time series ordinary least-squares regression output at 4 May 2020 intervention point (period: 9 March 2020–2 November 2020).

	β-Coefficient	Std Error	*p* Value	95% CI
Preintervention ^1^	−0.033	0.028	0.24	−0.089–0.023
Immediately postintervention ^2^	−2.05	0.875	0.02	−3.774–−0.326
Postintervention ^3^	0.049	0.029	0.09	−0.008–0.105
_cons ^4^	3.698	1.073	0.001	1.585–5.811
Postlinear trend regression output from 4 May 2020 intervention point.				
	β-Coefficient	Std error	*p* value	95% CI
Postintervention linear trend	0.016	0.005	0.002	0.006–0.025

^1^ Slope prior to intervention. ^2^ Change in level in the period immediately following intervention initiation (compared with counterfactual).^3^ Difference between preintervention and postintervention slopes. ^4^ Intercept.

**Table 5:** Interrupted time series ordinary least-squares regression output at 3 November 2020 intervention point (period: 4 May 2020–27 March 2021).

	β-Coefficient	Std Error	*p* Value	95% CI
Preintervention ^1^	0.016	0.005	0.002	0.006–0.025
Immediately postintervention ^2^	3.533	0.763	<0.0001	2.033–5.035
Pre- vs. postintervention ^3^	−0.051	0.008	<0.0001	−0.066–−0.035
_cons ^4^	−0.2	0.477	0.68	−1.138–0.738
Postlinear trend regression output from 3 November 2020 intervention point.				
	β-Coefficient	Std error	*p* value	95% CI
Postintervention linear trend	−0.035	0.006	<0.0001	−0.047–−0.023

^1^ Slope prior to intervention. ^2^ Change in level in the period immediately following intervention initiation (compared with counterfactual). ^3^ Difference between preintervention and postintervention slopes. ^4^ Intercept.

**Table 6:** Interrupted time series ordinary least-squares regression output at 28 March 2021 intervention point (period: 3 November 2020–31 December 2021).

	β-Coefficient	Std Error	*p* Value	95% CI
Preintervention ^1^	−0.035	0.006	<0.0001	−0.047–−0.023
Immediately postintervention ^2^	−1.341	0.511	0.009	−2.345–−0.337
Pre- vs. postintervention ^3^	0.042	0.006	<0.0001	0.029–0.054
_cons ^4^	6.194	0.545	<0.0001	5.122–7.266
Postlinear trend regression output from 28th March 2021 intervention point.				
	β-Coefficient	Std error	*p* value	95% CI
Postintervention linear trend	0.007	0.001	<0.0001	0.004–0.009

^1^ Slope prior to intervention. ^2^ Change in level in the period immediately following intervention initiation (compared with counterfactual). ^3^ Difference between preintervention and postintervention slopes. ^4^ Intercept.

## Data Availability

Anonymized data regarding the daily swab positivity ratio are available upon reasonable request.

## Supplementary Materials

The following supporting information can be downloaded at: https://www.mdpi.com/article/10.3390/vaccines10111847/s1. Figure S1: Staff population distribution according to their role (percentages are calculated over the period 3 January 2020–2 September 2022); Table S1: Staff population and PPE supply according to risk stratification and availability.
