# Supplementary material for: Effect of Lockdowns on Hospital Staff in a COVID Center: A Retrospective Observational Study
Source: Vaccines (Basel). 2022 Oct 31;10(11):1847. doi: 10.3390/vaccines10111847 (PMC9698425; doi:10.3390/vaccines10111847)

Staff population and PPE supply according to risk stratification and availability

	01.03.2020-31.03.2020	01.04.2020-05.11.2020	06.11.2020-31.12.2021	01.01.2022-09.02.2022
Healthcare Workers (medical staff, nurses, allied health professions)	**COVID DEPTs:** Surgical mask + Contact and droplet PPE	**COVID DEPTs:** Surgical mask OR FFP2 mask + Contact and droplet PPE	**COVID DEPTs:** FFP2 mask + Contact and droplet PPE	**COVID DEPTs:** FFP2 mask + Contact and droplet PPE
	**Other DEPTs:** Standard measures (social distancing, hand hygiene, etc.)	**Aerosol-producing procedures:** FFP2 mask or higher + Contact and droplet PPE	**Aerosol-producing procedures:** FFP3 mask + Contact and droplet PPE	**Aerosol-producing procedures:** FFP3 mask + Contact and droplet PPE
		**Other DEPTs:** Surgical mask + Standard measures	**Other DEPTs:** Surgical mask* + Standard measures	**Other DEPTs:** FFP2 + Standard measures
Administrative staff<br>Support staff<br>Other (carpenters, cafeteria workers, etc.)	Standard measures (social distancing, hand hygiene, etc., including smart working if applicable)	Surgical mask + Standard measures	Surgical mask + Standard measures	Surgical mask + Standard measures

** FFP2 mask in case of performing swabs or in case of ascertained clusters*

Staff population distribution according to their role (percentages are calculated over the period 01.03.2020-09.02.2022)

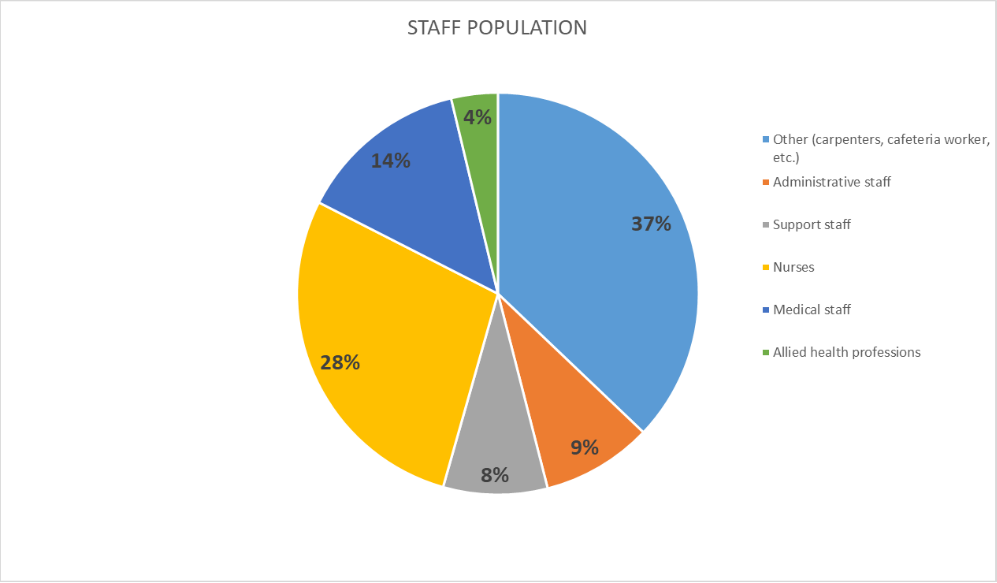

Supplement: Supplementary file 1 [file vaccines-10-01847-s001.zip › vaccines-1960578-supplementary.pdf]
